# New highly antigenic linear B cell epitope peptides from *Pv*AMA-1 as potential vaccine candidates

**DOI:** 10.1371/journal.pone.0258637

**Published:** 2021-11-02

**Authors:** Raianna F. Fantin, Vanessa G. Fraga, Camila A. Lopes, Isabella C. de Azevedo, João L. Reis-Cunha, Dhelio B. Pereira, Francisco P. Lobo, Marcela M. de Oliveira, Anderson C. dos Santos, Daniela C. Bartholomeu, Ricardo T. Fujiwara, Lilian L. Bueno

**Affiliations:** 1 Department of Parasitology, Federal University of Minas Gerais, Belo Horizonte, Minas Gerais, Brazil; 2 School of Medicine, Federal University of Minas Gerais, Belo Horizonte, Brazil; 3 Department of Preventive Veterinary, Medicine Federal University of Minas Gerais, Belo Horizonte, Brazil; 4 Tropical Medicine Research Center, Porto Velho, Rondônia, Brazil; Instituto Butantan, BRAZIL

## Abstract

Peptide-based vaccines have demonstrated to be an important way to induce long-lived immune responses and, therefore, a promising strategy in the rational of vaccine development. As to malaria, among the classic vaccine targets, the Apical membrane antigen (AMA-1) was proven to have important B cell epitopes that can induce specific immune response and, hence, became key players for a vaccine approach. The peptides selection was carried out using a bioinformatic approach based on Hidden Markov Models profiles of known antigens and propensity scale methods based on hydrophilicity and secondary structure prediction. The antigenicity of the selected B-cell peptides was assessed by multiple serological assays using sera from acute *P*.*vivax* infected subjects. The synthetic peptides were recognized by 45.5%, 48.7% and 32.2% of infected subjects for peptides I, II and III respectively. Moreover, when synthetized together (tripeptide), the reactivity increases up to 62%, which is comparable to the reactivity found against the whole protein *Pv*AMA-1 (57%). Furthermore, IgG reactivity against the tripeptide after depletion was reduced by 42%, indicating that these epitopes may be responsible for a considerable part of the protein immunogenicity. These results represent an excellent perspective regarding future chimeric vaccine constructions that may come to contemplate several targets with the potential to generate the robust and protective immune response that a vivax malaria vaccine needs to succeed.

## Introduction

Malaria disease represents a huge challenge for public authorities in Brazil and in the worldwide. Of the species that infect humans, *Plasmodium vivax* and *Plasmodium falciparum* are considered to be the most important from a public health point of view. While deaths and several cases are mostly reported coming from *P*. *falciparum infection*, *P*. *vivax* is responsible for causing 90% of cases registered outside Africa. In 2019, an estimated 229 million cases of malaria occurred worldwide [1]. Specifically in Brazil, 194,000 cases were reported in the same period and *P*. *vivax* stands out being responsible for around 80% of those [2]. This species is particularly challenging for malaria control not only because of its wider global distribution but also because of its high frequency of sub-microscopic infections, and ability to produce relapses from long-lasting liver-stages infections [3, 4].

In the last decades, efforts have been made to develop vaccines against different stages of *P*. *vivax*. Among the classic targets, the Apical membrane antigen (AMA-1), a transmembrane protein located in the micronemes of the parasite and present in all species of *Plasmodium*, is a promising target to induce a protective immune response as demonstrated in rodent and non-human primates’ experimental models [5]. Among its functional aspects, the mediation of parasite internalization within the host cell through a structure formed by AMA-1 and the erythrocyte complex of proteins named Rhoptry Neck (RON), is one of the most remarkable [6–8]. There is strong evidence that antibodies and peptides against the junction formed by these two molecules can inhibit the invasion of parasites in the erythrocytes of human hosts [5, 9]. Furthermore, specifically regarding *P*. *vivax* AMA-1 (*Pv*AMA-1), immuno-epidemiological studies conducted in different endemic regions of Brazil, India and Sri-Lanka have demonstrated a naturally acquired immune response to this protein even in individuals with limited exposure to the disease [10, 11]. Of note, a linear epitope derived from the domain II of *Pv*AMA-1 was identified through bioinformatic analysis and synthetic production [7] and the favorable data support for the development of a *Pv*AMA-1-based subunit vaccine against malaria vivax infection. Indeed, the identification of highly immunogenic peptides through bioinformatic tools is one of the most promising [12, 13]. Developing peptide-based antigens may have numerous advantages. Amidst them, the most important ones navigate through cost, stability, easy reproducibility, capacity in result specific immune response and the possibility to generate chimeras containing multiple epitopes that might be relevant [14]. Moreover, antigens that are peptide-based have demonstrated to be able to induce long-lived immune responses since they can be customized to aim very specific targets [15].

In this context, the identification of immunodominant targets that allows the development of vaccines against *P*. *vivax* may be critical in the success of a research agenda to underpin malaria control and elimination. Here we present three new highly antigenic linear B cell epitope peptides within the *Pv*AMA-1 as potential vaccine candidates. They have showed to be strong candidates when assessed individually or as a multi-component antigen, with an immunogenicity that is equivalent to the entire *Pv*AMA-1 protein.

## Methods

### Study population, sera samples and ethical statements

The study population included 121 individuals living in the Brazilian Amazon area who were diagnosed with vivax malaria at the Research Center for Tropical Medicine (CEPEM) in Porto Velho, Rondônia, Brazil, in the years of 2014 (Jul) and 2019 (Nov–Dec). The volunteer’s inclusion criteria in this study group was (i) to have an acute infection at the moment of blood sampling; (ii) be over 18 years of age and (iii) to be willing to participate in the study. Considering that the subjects participating in the study were chosen as they sought the city’s health service, the selection was random, in a way that allowed each member of the larger group—the population of Porto Velho—to have an equal chance of being chosen. Although certain characteristics such as occupation and place of residence have been shown to be of importance in the rate of infection in other studies [16–18], since certain environments can lead to greater exposure to the vector (farmers, rural households, etc.), in this study no relationship was observed between occupation/residence and malaria incidence. Fifteen healthy individuals from Belo Horizonte, Minas Gerais State, Brazil, a non-endemic area for malaria, were also recruited as controls. Each volunteer was required to sign a written informed consent specific to this study, and blood was obtained upon receiving the said document. The research was approved by the Research Ethics Council of the Federal University of Minas Gerais, Brazil (CAAE 27466214.0.0000.5149).

### Peptide selection

The prediction of B cell linear epitopes was carried out using the program BepiPred 1.0 as described before [10]. Briefly, the software takes a single sequence in FASTA format as input, and each amino acid receives a prediction score based on Hidden Markov Models profiles of known antigens and incorporates propensity scale methods based on hydrophilicity and secondary structure prediction. The lowest cut-off of 0.35 was used in order to consider a given region as a valid linear B cell epitope. The epitope score represents the average of the scores of individual amino acids above the cut-off.

#### Peptide conservation evaluation

To evaluate the conservation of the selected peptides, a total of 218 *Pv*AMA-1 sequences were recovered from PlasmoDB [19]. Out of those, only sequences with a read depth coverage higher than 30 and with at least 90% of its reads mapping to the reference were selected, using the metadata provided by PlasmoDB. Next, sequences that had 10 or more “N”s were removed, using in-house Perl scripts, resulting in a final selection of 64 sequences for downstream analysis. These 64 sequences were aligned using MAFFT v7.427 [20], with—maxiterate 1000—globalpair—adjustdirection–reorder options. The aligned sequences were then translated with SeqKit translate v.0.12.0 [21], and the conservation estimation, plots, tables, and logo images were generated in R (https://www.R-project.org/), using the following libraries: seqinr [22], ggplot2, and ggseqlogo [23].

### Synthesis and mass spectrometry of soluble peptides

The soluble peptides EEFRDYYENGEEKSNKQM (PI), SSGVRVDLGEDAEVENAK (PII), GDQRLKDGGFAFPNADDH (PIII) and KLKDGGFAFPNANDHEEFRDYYENGEEKSNKQM (tripeptide) were synthesized on a 25 μmol scale in the ResPep SL automated synthesizer (Intavis^®^). Brielfy, Fmoc-amino acids were activated with a 1∶1 solution of Oxyma Pure (Merk) and diisopropylcarbodiimide (DIC, Sigma-Aldrich). The active amino acids were incorporated into TentaGel (Intavis) (Peptides I and II) or H-Rink Amide ChemMatrix (Sigma-Aldrich) (Peptide III and Tripeptide) resins. Fmoc deprotection was performed using 25% 4-methylpiperidine (25% v/v in DMF). These steps were repeated until the synthesis of each peptide was completed. The peptides were deprotected and released form the resin by treatment with a solution of 92.5% trifluoroacetic acid, 2.5% water, 2.5% triisopropylsilane and 2.5% beta-mercaptoethanol during 3 hours under agitation. Specifically, for the Tripeptide the synthesis was performed using a chaotropic agent and lithium chloride (0.8M). The time of deprotection was prolonged in order to guarantee the proper extension of the polypeptide chain. The peptides were precipitated with cold methyl tert-butyl ether and lyophilized. The molecular weight of each peptide synthetized was confirmed by mass spectrometry using Autoflex Speed MALDI/TOF equipment.

### Mass spectrometry (MALDI/TOF)

For the analysis, 0.5 μL of the concentrated sample was mixed with 0.25 mL of a saturated matrix solution 10 mg/mL α-cyano-4-hydroxycinnamic (Aldrich, Milwaukee, WI) in 50% acetonitrile/0.1% trifluoroacetic acid. The samples were applied to a MTP AnchorChip ™ 600/384 plate (Bruker Daltonics) and left to dry at room temperature. The raw data was obtained by MALDI-TOF/TOF Autoflex III™ (Bruker Daltonics, Billerica, USA) using a positive/reflector mode controlled by FlexControl™ 3.3 software. The instrument calibration was performed using reference peptides (Peptide Standard, Bruker Daltonics). Each spectrum was produced by accumulating data from 200 consecutive laser shots.

### Expression and purification of the Apical membrane Protein 1 (*Pv*AMA-1/Sal-1)

The protein representing amino acids 1 to 546 of AMA-1 was expressed in *Escherichia coli*, as previously described [10, 24]. Briefly, the recombinant plasmid containing pUC57/AMA-1 synthetic gene (GenScript) was resuspended and transformed with competent cells from *Escherichia coli* XL-1 Blue (Phoneutria, Brazil). After positive clones were confirmed by digestion with the enzymes *XhoI* and *NheI*, the AMA-1 insert was subcloned into a bacterial expression vector pET28aTEV. Electrocompetent *E*. *coli* BL21-Star (Thermo Fisher Scientific, USA) cells were transformed with the recombinant plasmid *p*ET28a-TEV/AMA-1 by electroporation using a MicroPulser Electroporation Apparatus (Bio-Rad Laboratories, USA). Correct gene insertion was confirmed by colony PCR and using T7 primers (Macrogen, South Korea). Protein large scale expression was obtained after the addition of 1mM isopropyl β-D-1-thiogalactopyranoside (IPTG) and incubation for 3 h at 37°C at 180rpm. Cells were ruptured by a high-pressure homogenizer and soluble fractions were obtained by centrifugation. The recombinant protein was purified using Ni^2+^ affinity chromatography with HisTrap HP 5 mL column (GE Healthcare, USA) coupled to an ÄKTA Prime Plus system (GE Healthcare, USA). The purified AMA-1 protein, having 546 amino acids and predicted molecular weight of 62kDa, was separated by SDS-PAGE.

### Serological assays

#### Total IgG

The presence of IgG antibodies against *Pv*AMA-1 protein and all four peptides were determined through a conventional enzyme-linked immunosorbent assay (ELISA) that was performed as previously described [10, 25]. Briefly, the microplates (Costar®) were coated with the antigens (concentrations of 0.5μg/mL and 2μg/well for *Pv*AMA-1 and peptides respectively) and incubated overnight at 4°C for *Pv*AMA-1 and at 37°C for peptides. Subsequently, the plates were washed and blocked (PBS 1x pH 7.2 + 3% BSA). All samples were diluted 1:100 and evaluated for total IgG using peroxidase-conjugated anti-human IgG antibodies at a concentration of 1:10.000 (Catalog No. W4031) (Promega Corporation). Monoclonal antibody binding was detected with OPD substrate tablets (Thermo Fisher Scientific, USA). The *cut-off* value was obtained by testing 15 different negative control sera from individuals never exposed to malaria from Belo Horizonte, Brazil. The mean optical density value at 492 nm ± 3 SD (VersaMax^TM^ Microplate Reader) for duplicate determinations in negative sera was used as the cut-off value for different peptides.

#### IgG subclasses

The ELISA to detect the IgG subclasses was performed as previously described [10]. The sera were diluted 1:100 and evaluated for each IgG subclass using mouse monoclonal antibodies to human IgG subclasses (IgG1 clone 8c/6-39; IgG2 clone HP-6014; IgG3 clone HP-6050; IgG4 clone HP-6025) (Sigma, St. Louis, MO) according to the manufacturer instructions. Specifically, the secondary antibody dilutions were 1:1.000, 1:15.000, 1:40.000 and 1:60.000 for IgG 1, IgG 2, IgG 3 and IgG 4 respectively. The *cut-off* value was obtained by testing 6 different negative control sera from individuals never exposed to malaria from Belo Horizonte, Brazil. Monoclonal antibody binding was detected with OPD substrate tablets (Thermo Fisher Scientific, USA). The mean optical density value at 492 nm±3 SD (VersaMax^TM^ Microplate Reader) for duplicate determinations in negative sera was used as the cut-off value for different subclasses and peptides.

### Depletion ELISA

The depletion ELISAs were performed as previously described [26]. Briefly, flat-bottom plates (Costar, USA) were coated overnight with 2 μg/well of the peptides EEFRDYYENGEEKSNKQM (PI), SSGVRVDLGEDAEVENAK (PII), GDQRLKDGGFAFPNADDH (PIII) and SSGVRVDLGEDAEVENAKGDQKLKDGGFAFPNANDHEEFRDYYENGEEKSNKQM (tripeptide), then washed and blocked as described. Sera were added to the plates at a 1:100 dilution and incubated at 37°C overnight. On the following day, sera were transferred to plates coated overnight with *Pv*AMA-1 (0,5 ug/mL) after appropriate washing and blocking.

### Statistical analysis

All statistics were carried out using Graphpad Prism 8 for iOS (Graphpad Software, Inc.). In order to determine whether a variable was normally distributed, we used Shapiro-Wilk and Kolmogorov-Sminorff tests. The frequency and association between IgG antibody response (total and subclasses) to *Pv*AMA-1 and all four peptides was determined by Fisher’s exact test. P values for the depletion ELISA were determined by Wilcoxon matched pairs test. All p values <0.05 were considered significant.

## Results

### Epidemiological profile of studied individuals

The study population is composed of 121 individuals residing in the city of Porto Velho (Rondônia) and neighboring municipalities (Table 1). The majority is composed of adult individuals naturally exposed to the malaria parasite and the age range was 29–- 49 with an average of 39 years old. The gender ratio was 1:3 (female: 23.9%; male: 76.1%). Regarding previous malaria infections, the majority of them have had both most prevalent species currently circulating in Brazil: *P*. *falciparum* and *P*. *vivax* (44.6%), following by those who have only been infected with *P*. *vivax* (37.2%); with none of them (15.7%) and finally, only with *P*. *falciparum* (1.65%). In general, the parameters, that might differ depending on gender and occupation, indicate that individuals are frequently exposed to malaria. The control population was comprised of 15 non-exposed and never infected individuals living in a non-endemic area. These individuals had an age range of 26.5–33, with an average of 28 years. The gender ratio was (female: 61.53% male: 38.46%) (Table 2).

**Table 1:** Epidemiological and demographic characterization of infected subjects from endemic area (2014/2019), n = 121.

Median age, years (IQR)^a^	39 (28–49)
**Gender, female:male**	1:3
**% of individuals previously infected with malaria**	86.8% (105)
**Previous malaria episodes, median (IQR)**	5 (2–11)
*Plasmodium vivax*	37.2% (45)
*Plasmodium falciparum*	1.65% (2)
***P*.*vivax* and *P*.*falciparum***	44.6% (54)
**None**	15.7% (19)

Epidemiological and demographic characteristics of the 121 individuals selected for the study from a malaria endemic region (Porto Velho, Rondônia, Brazil).

^a^IQR: Interquartile range.

**Table 2:** Epidemiological and demographic characterization of non-infected subjects/controls from non-endemic area (2019), n = 15.

Median age, years (IQR)^a^	28 (26.5–33)
**Gender, female:male**	1.6:1
**% of individuals previously infected with malaria**	0 (0)
**Previous malaria episodes, median (IQR)**	0 (0)

Epidemiological and demographic characteristics of the 15 individuals selected for the study from a malaria free area (Belo Horizonte, Minas Gerais, Brazil).

^a^IQR: Interquartile range.

### Prediction results, chemical synthesis, and quality assessment of selected peptides

**Fig 1:**
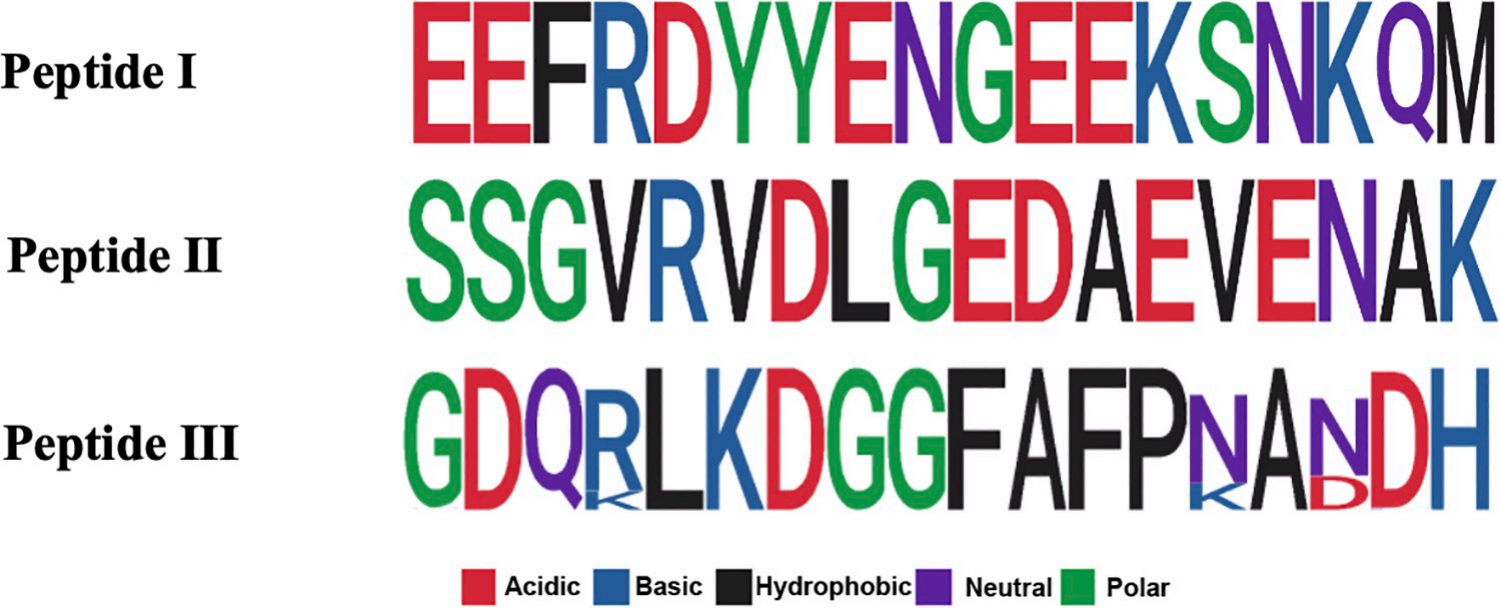
Logo representation of the amino acid conservation of the selected peptides in 64 P. vivax isolates from PlasmoDB. The frequency of each amino acid in each position is represented by the size of its corresponding letter. Each colour corresponds to a physicochemical property.

**Fig 2:**
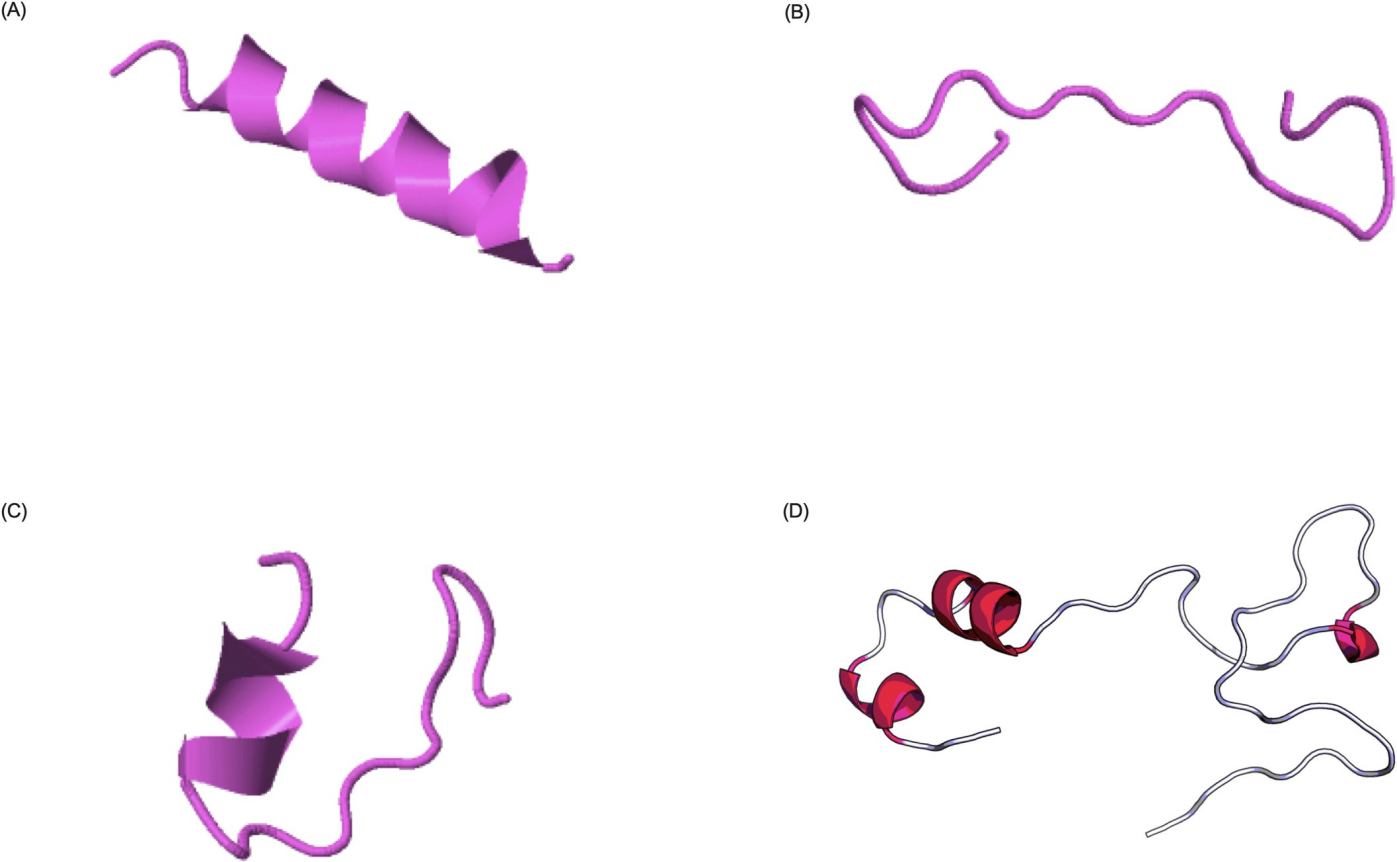
Predicted secondary structure of the synthetic peptides. (A) Peptide I (EEFRDYYENGEEKSNKQM): helix and coil dominance. (B) Peptide II (SSGVRVDLGEDAEVENAK): Strand and coil dominance. (C) Peptide III (GDQRLKDGGFAFPNADDH): coil dominance. (D) Tripeptide (SSGVRVDLGEDAEVENAKGDQKLKDGGFAFPNANDHEEFRDYYENGEEKSNKQM): helix and beta dominance.

The predicted peptides were found to be distributed within two of the three *Pv*AMA-1 domains, being PI part of domain III, and P II and III part of domain I. An analysis conducted using PlasmoDB sequences confirmed that the peptides are well conserved (Fig 1), with little or no variation in amino acids positions (S1 Fig, S1 Table), especially PI, which demonstrated to be 100% conserved in all assessed isolates. The molecular weights (MW) of each specie were accessed in PepCalc.com–- Peptide calculator (Innovagen^TM^) namely: PI (2295.4 g/mol), PII (1873.97 g/mol), PIII (1930.04 g/mol) and Tripeptide (6065.35 g/mol). All MW were experimentally confirmed through characterization of the synthetic peptides by MALDI-TOF-TOF (S2 Fig). It is possible to observe the isotopic envelope of the small molecules (PI, PII and PIII) in the graphs (S2A–S2C Fig respectively), since it was able to use the reflector mode in their characterization. It wasn’t possible to observe the isotopic envelope of the largest species–tripeptide–whereas the linear mode with lower resolution was used in the characterization, considering the reduced ionization capacity of this molecule. The tridimensional structure of each peptide was predicted using two different software: i-TASSER for smaller peptides (Fig 2A–2C) and RaptorX for the bigger one, as shown in Fig 2D [27–31]. We observed that PI and PII exhibits some regions in α-helix despite their small sizes and their secondary structures are partially preserved in the Tripeptide.

### Evaluation of total IgG response to selected antigens

**Fig 3:**
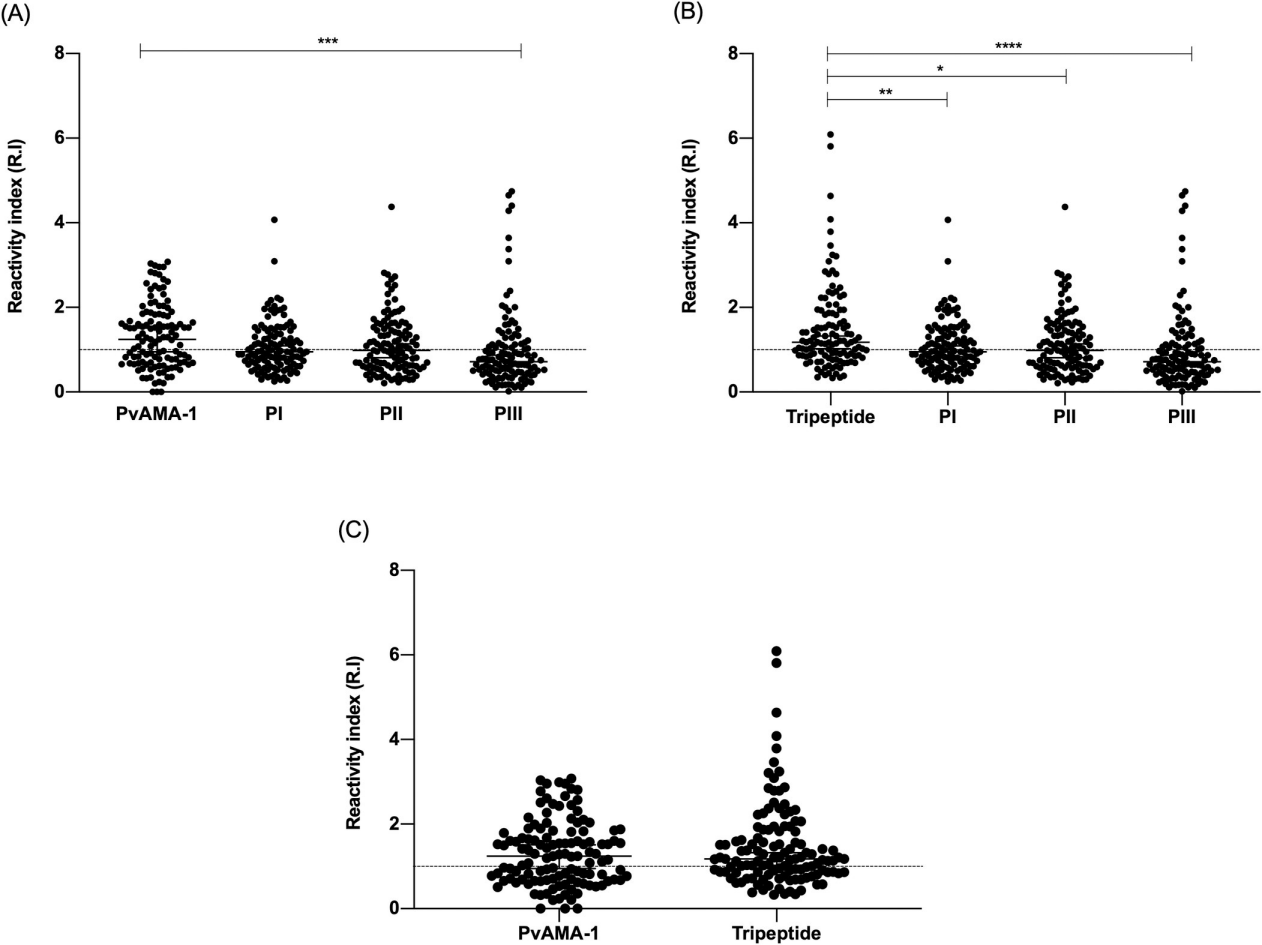
Levels of specific IgG antibodies to *Pv*AMA-1, Peptide I (PI), Peptide II (PII), Peptide III (PIII) and tripeptide. (A) Comparison of the IgG levels between *Pv*AMA-1 and the 18aa peptides. (B) Comparison of the IgG levels between tripeptide and the 18aa peptides. (C) Comparison between *Pv*AMA-1 and tripeptide. The Y axis represents the mean reactivity index in naturally *P*. *vivax* infected individuals (= 121). The dotted line shows the positivity threshold (reactivity index = 1). The data were analyzed considering a 95% confidence interval (CI). P < 0.05 was considered significant.

In the current study, the presence of total IgG antibody response from naturally *P*. *vivax*-infected individuals was assessed in face of the selected peptides (18 and 54 amino acids) and the whole *Pv*AMA-1 (560 amino acids) in order to compare the immunogenic capacity of each of them. For this purpose, samples from 121 individuals with acute infection by *P*.*vivax* were evaluated by conventional serology (ELISA). Regarding the frequency of individuals responding to the tested peptides and proteins, we observed a 57% positivity for *Pv*AMA-1 (69/121), 62% for the tripeptide (76/121) and 45.5% (55/121), 48.7% (59/121), 32.2% (39/121) for peptides I, II and III respectively. When compared to peptides I, II, III, the *Pv*AMA-1 (Fig 3A) presents a higher and statistically significant frequency only when compared to peptide III (p = <0.0002 Fisher’s exact test). Regarding peptides I and II, there is no significant difference between the frequency of responses, which suggests that those peptides alone can be recognized as much as the whole protein *Pv*AMA-1 (PI p = 0.09; PII p = 0.24, Fisher’s exact test). The same cannot be observed when we compare the frequency of response of the tripeptide (54aa) to the smaller ones. The tripeptide, which represents the chemical junction of peptides I, II and III, shows a higher frequency of positivity in all comparisons (PI p = 0.009; PII p = 0.038; PIII p = <0.0001, Fisher’s exact test) (Fig 3B). Of relevance, although no significant differences were observed between the immunogenic capacity of *Pv*AMA-1 and the tripeptide (p = 0.43%, Fisher’s exact test), the frequency of respondents was different between these two antigens (Fig 3C). More specifically, the percentage of individuals with specific antibodies to the tripeptide was slightly higher than against the whole protein (+ 5%).

### Similar IgG subclass response pattern between tested peptides

**Fig 4:**
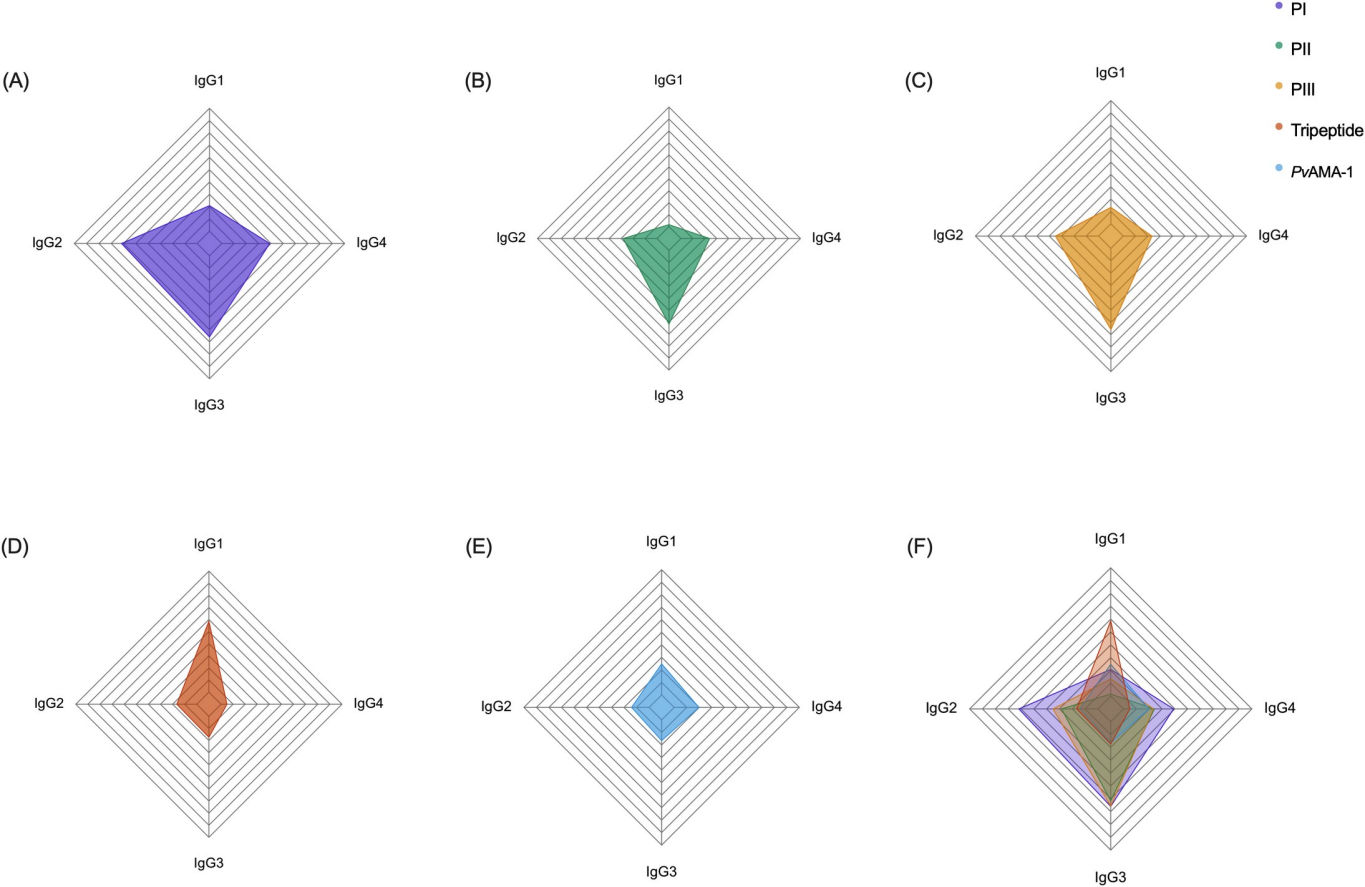
Proportion of IgG subclasses antibodies to the synthetic peptides and PvAMA-1. (A) Peptide I; (B) Peptide II; (C) Peptide III; (D) Tripeptide; (E) PvAMA-1; (F) Peptides and Pv-AMA-1 merged together. The radar charts are divided into ten lines, each of them representing a 10% value. On each radar chart, data is shown as the frequency of individuals responding to an IgG subclass (n = 121). The data were analyzed considering a 95% confidence interval (CI). P < 0.05 was considered significant.

Although this type of data may considerably vary among individuals naturally infected with *Plasmodium vivax*, the level of specific antibodies for each IgG subclass followed a pattern as for peptides composed of only 18 amino acids (PI, PII, PIII). For them, there was a higher frequency of IgG2 (PI: 61.6% PII: 28.30% PIII: 35%) and IgG3 (PI: 66%; PII: 61.6%; PIII: 65.8%) (Fig 4A–4C). When compared to *Pv*AMA-1, there was a statistically significant difference for the following subclasses: IgG 2 (PI p = <0.0001; PII p = 0.01; PIII p = 0.0003, Fisher’s exact test); IgG 3 (PI, PII and PIII p = <0.0001, Fisher’s exact test); IgG 4 (PI p = 0.001, Fisher’s exact test). As for the tripeptide and *Pv*AMA-1, subclass 1 had more frequent and robust recognition (Fig 4D and 4E).

When compared, the frequencies showed by both antigens (*Pv*-AMA-1 and tripeptide) have shown statistic similarity, except for subclass 1 (IgG 1) in which, for the tripeptide, we have 58.6% of recognition against 24.8% for *Pv*AMA-1 (p ≤0.0001, Fisher’s exact test). Overall, the *Pv*AMA-1 protein showed lower frequencies when compared to the four peptides, although in some cases the difference is not statistically significant (Fig 4F).

### Peptide immunodominance within total IgG antibody response

**Fig 5:**
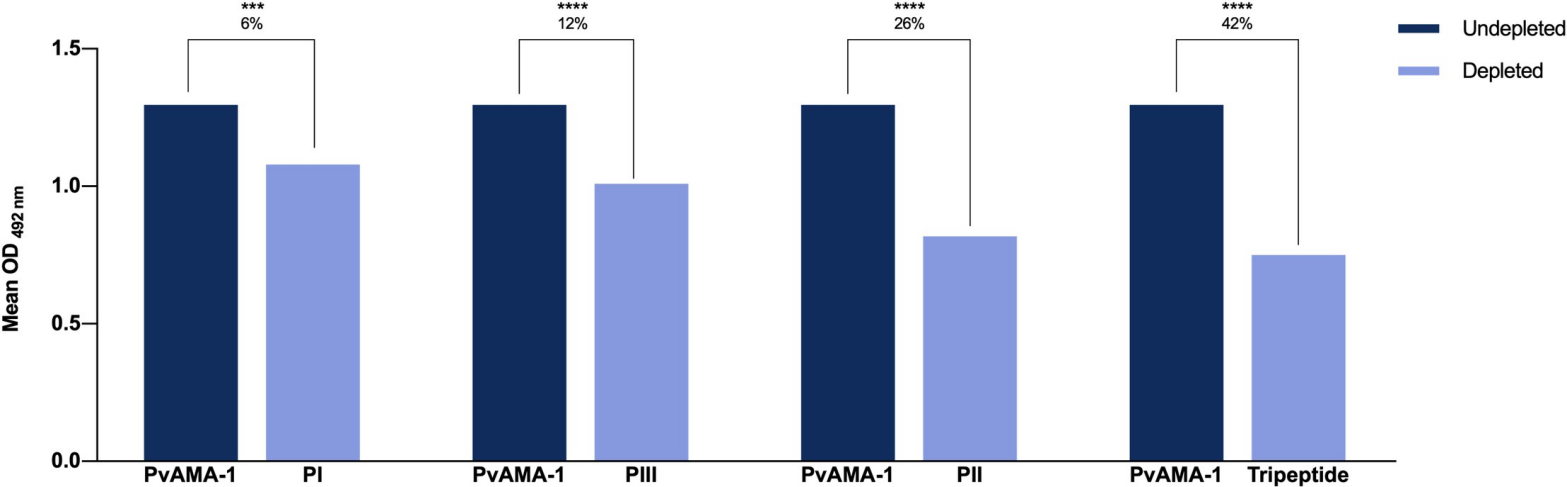
Immunodepletion showing specific antibody recognition to the synthetic peptides (PI, PII, PIII and tripeptide) in naturally *P*. *vivax*-infected individuals. The sera were depleted with all four synthetic peptides and then evaluated for *Pv*AMA-1. The Y-axis represents the mean antibody OD values (n = 121). The data were analyzed considering a 95% confidence interval (CI). P < 0.05 was considered significant.

In order to determine the immunodominance of each peptide, a depletion ELISA assay was performed. In this assay, reactivity against *Pv*AMA-1 (IgG) was reduced substantially for all tested peptides (Fig 5). Respectively, the reduction was 6%, 12%, 26% and 42% for PI, PIII, PII and tripeptide peptides.

## Discussion

Although it has been available for a considerable time, *P*. *vivax* genome and proteome has been little explored in terms of identifying vaccine targets. Accordingly, the search using computational analysis is a major draw, since it has been shown to be highly effective in identifying targets within *Plasmodium* species, in addition to providing optimization of research time and vaccine production [32]. Here in, we evaluated the sero-immunological response of individuals from the Brazilian Amazon naturally infected by *P*. *vivax* against peptide targets identified within an important and essential protein: *Pv*AMA-1. The individuals profile included in the study indicates that all of them are residents of an endemic area for malaria. Most individuals reported having been infected previously (86.8%) and the large variation between the number of previous episodes, as well as the predominance of males among those affected, is most likely due to different levels of exposure that may be related to the personal occupation of each individual.

The major goal was to assess the immunogenic capacity of selected targets using *in silico* techniques and to compare it with the response obtained from the main protein—*Pv*AMA-1—which is currently one of the main vaccine targets for malaria vivax [33, 34]. Data from previous studies carried out with rodents and non-human primates have shown that *Pv*AMA-1 has already proved to be a promising target for inducing a protective immune response [10, 11, 35]. Several of them have confirmed its immunogenicity, especially in endemic areas such as the Brazilian Amazon [36–38]. In addition, the functionality of AMA-1-related antibodies has also been assessed. This protein was proved to be a key component in the invasion process through the moving junction (MJ) along with the RON complex, in which anti-AMA-1 antibodies were able to inhibit the binding interaction between them [10, 11, 35, 39, 40]. In our assessment, we found that the selected peptides can be as immunogenic as the protein from which they were extracted. While for *Pv*AMA-1 we have a total IgG response frequency of 57%, for subunits we found frequencies that varied between 32% - 62%, the largest of which came from the formulation containing the junction of the three peptides. In fact, with regard to *Pv*-AMA1, the response levels found corroborate with previous studies that evaluated the proteins immunogenic capacity [10, 41–43].

Among the disadvantages in peptide-based vaccine constructions, low immunogenicity is the most relevant. When compared to conventional vaccine formulations, such as those using inactivated or attenuated pathogens, the immune response generated can be considerably weaker [44, 45]. This is a relevant factor since a robust activation usually characterizes greater protection and longevity of the immune response, two very desirable vaccine features. However, stronger adjuvants may overcome this obstacle. A few peptide-based vaccines for wide-impact diseases like leukemia [46], secondary progressive multiple sclerosis [47], and, more recently, SARS-Cov2 [48], have reached phase III studies this way. The latter has demonstrated efficacy of approximately 90% and is currently in the process of being approved for emergency use worldwide [49, 50]. Regarding to the tested targets, this does not appear to be an important limitation since the response obtained by three of the four peptides does not differ from the one induced by the entire protein. This data can be relevant when comparing the sizes of all antigenic targets (546aa, 54aa and 18aa for *Pv*AMA-1, tripeptides and small peptides respectively) and considering the possibility of inserting the peptides in a chimeric construction containing multiple targets, which can generate an even more efficient response. Regarding to the response from the IgG subclasses, we found a similar pattern among small peptides with predominance of classes 2 and 3, highlighting the subclass 3. Individuals with high frequencies of IgG3 antibodies are known to have low parasitemia with clinical manifestations ranging from mild to moderate. For the tripeptide, a higher frequency of subclass 1 was observed. These data indicate that, in general, there is a dominant presence of cytophilic antibodies against peptide-based formulations among the tested individuals. It is well known that antibodies that belong to this class have the ability to activate effector cells of the immune system that can, therefore, play a protective role. Although it is not common, the relatively high frequency of IgG2 antibodies has also been observed in similar studies [51]. A study conducted in Ghana showed that the frequency of IgG2 antibodies in patients from malaria-endemic regions increased according to the individuals’ age, indicating that this subclass may be involved in acquired immunity [52]. However, it is known that non-cytophilic antibodies, such as the case of this group (IgG2), when acting against the same epitope, can undermine the protective effect of the cynophiles ones [53]. Nevertheless, the effective role of each subclass of antibodies in mediating protection against peptide-based antigens remains to be clarified.

Finally, the depletion assays demonstrated that the individuals’ antibodies can be quite specific against the studied antigens, with a recognition percentage of 6%, 12%, 26% and 42% for PI, PIII, PII and tripeptide respectively. In general, the results demonstrate that the peptides contain antigenic regions of extreme importance for the recognition of *Pv*AMA-1, confirming its potential as possible targets for a chimeric composition of antigens. Of relevance, when tested against the serum of mice infected with *Plasmodium berghei*, the four peptides were 100% recognized (S3 Fig). The AMA-1 homology among the two species is around 50% [54] and, specifically amid the synthetic formulations, the amino acid overlapping can go from 33.4% up to 72.2% depending on the peptide. Whilst the common residues between the two of them may play a key role in antibody biding, further investigation on the matter, including an increase in the number of the P. *berghei*-infected subjects, is needed. Yet, this data is of considerable relevance since, in the future, a possible vaccine formulation will need to undergo studies in animal models and it is essential that there is homology in the recognition by the malaria species that are capable of infecting them.

## Conclusion

Based on in silico approaches for choosing potential targets, the present study exhibit three new peptide antigens with considerable immunogenic capacity when evaluated individually and quite expressive when under joint synthesis. The identification and initial assessment of these four linear B-cell epitopes represents an excellent perspective with regard to future chimeric vaccine constructions that may come to contemplate several targets with the potential to generate the robust and protective immune response that a vivax malaria vaccine needs to succeed.

## Supporting information

S1 Fig Amino acid variability of three PvAMA-1 isolates sequences, from PlasmoDB. The X-axis corresponds to the amino acid position, while the Y-axis corresponds to the number of variants observed in each position. Variants were defined when an amino acid did not match to the most commonly observed in a given position.
(TIF)

S2 Fig Characterization of the synthetic peptides by MALDI-TOF-TOF. The x-axis represents mass/load, while y-axis defines intensity (arbitrary unity). (A) Peptide I (EEFRDYYENGEEKSNKQM). (B) Peptide II (SSGVRVDLGEDAEVENAK). (C) Peptide III (GDQRLKDGGFAFPNADDH). (D) Tripeptide (SSGVRVDLGEDAEVENAKGDQKLKDGGFAFPNANDHEEFRDYYENGEEKSNKQM).
(TIF)

S3 Fig Levels of specific total IgG antibodies in mice experimentally infected with *Plasmodium berghei* (n = 6). Subjects with a Reactivity Index above 1 were considered positive. The R.I was calculated considering the mean OD of non-infected subjects (controls) +2SD (95% confidence interval).
(TIF)

S1 Table Presence of amino acids in each single position within *Pv*AMA-1. Rows corresponds to all amino acids belonging to PvAMA-1 (1–563) and columns represents a specific position within the protein (a—y). The numbers indicate how many times a given amino acid occurs at each position ranging from 0–64 times. Red boxes indicate regions corresponding to the peptides under study.
(XLSX)
