# Supplementary figures and images for: New highly antigenic linear B cell epitope peptides from PvAMA-1 as potential vaccine candidates
Source: PLoS One. 2021 Nov 2;16(11):e0258637. doi: 10.1371/journal.pone.0258637 (PMC8562794; doi:10.1371/journal.pone.0258637)

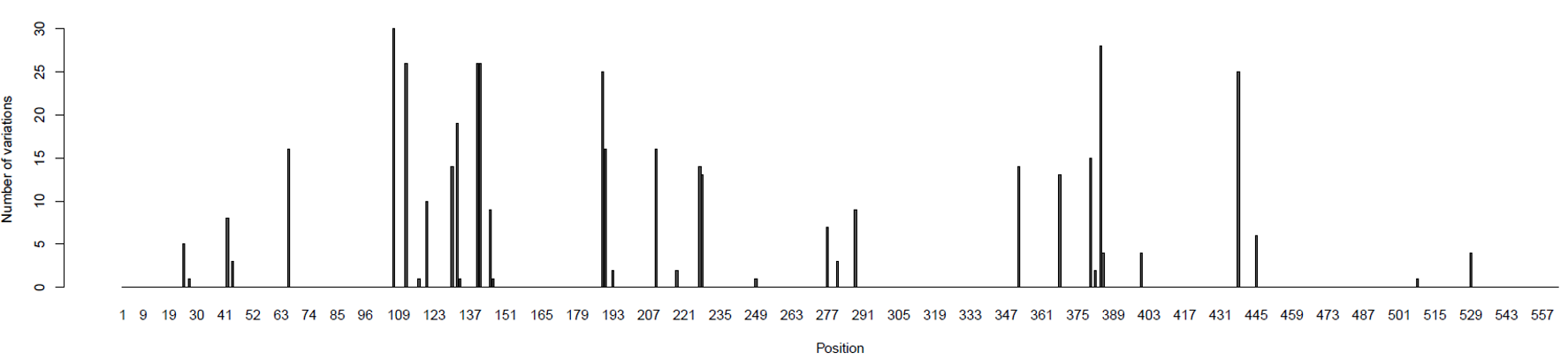

Supplement: S1 Fig — The X-axis corresponds to the amino acid position, while the Y-axis corresponds to the number of variants observed in each position. Variants were defined when an amino acid did not match to the most commonly observed in a given position. (TIF) [file pone.0258637.s001.tif]

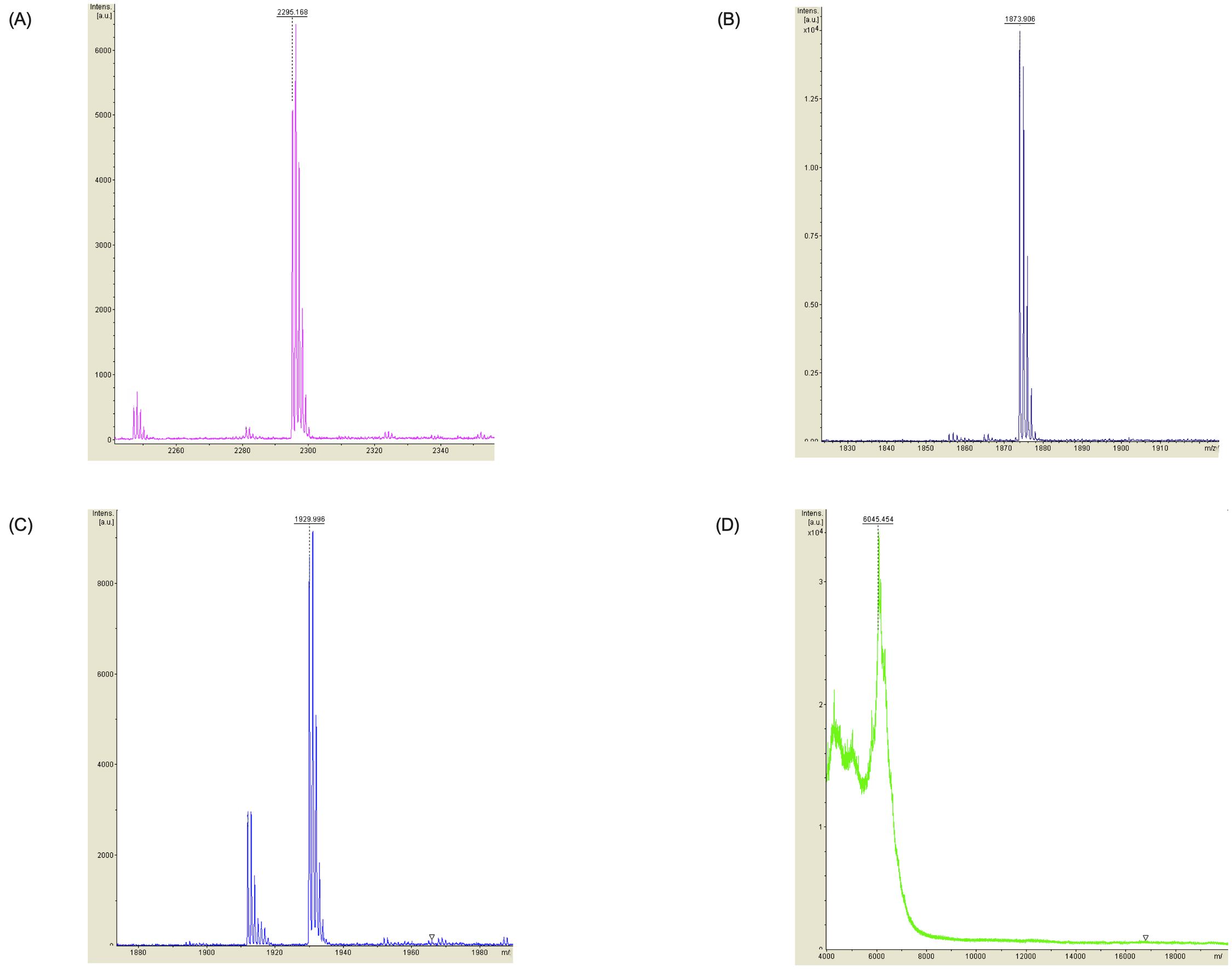

Supplement: S2 Fig — The x-axis represents mass/load, while y-axis defines intensity (arbitrary unity). (A) Peptide I (EEFRDYYENGEEKSNKQM). (B) Peptide II (SSGVRVDLGEDAEVENAK). (C) Peptide III (GDQRLKDGGFAFPNADDH). (D) Tripeptide (SSGVRVDLGEDAEVENAKGDQKLKDGGFAFPNANDHEEFRDYYENGEEKSNKQM). (TIF) [file pone.0258637.s002.tif]

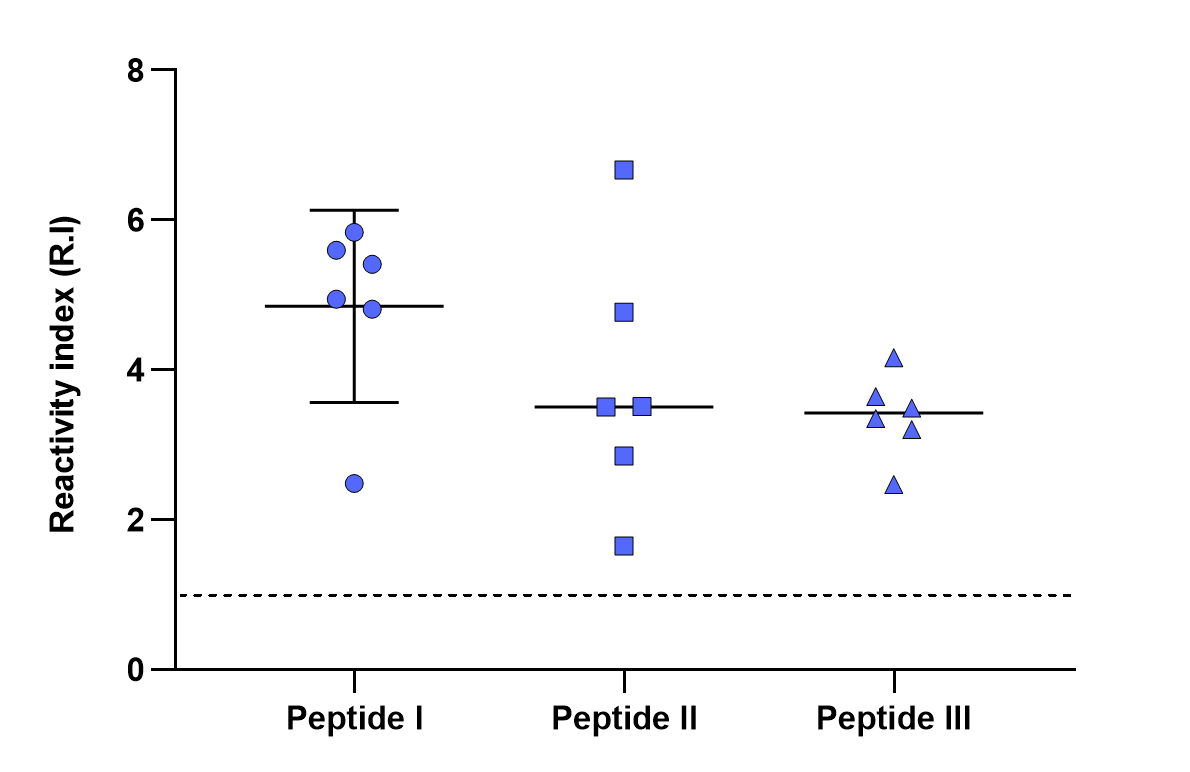

Supplement: S3 Fig — Subjects with a Reactivity Index above 1 were considered positive. The R.I was calculated considering the mean OD of non-infected subjects (controls) +2SD (95% confidence interval). (TIF) [file pone.0258637.s003.tif]
